# Immune gene signatures and tumor intrinsic markers delineate novel immunogenic subtypes of breast cancer

**DOI:** 10.1186/2051-1426-2-S3-P256

**Published:** 2014-11-06

**Authors:** Lance D Miller, Jeff W Chou, Michael A Black, Cristin G Print, Eric Jimenez, Julia Chifman, Angela Alistar, Thomas C Putti, Xiaobo Zhou, Davide Bedognetti, Ashok Pullikuth, Eran Andrechek, Ena Wang, Francesco M Marincola

**Affiliations:** 1 Wake Forest School of Medicine, Winston Salem, NC, USA; 2 Wake Forest Public Health Sciences, Department of Biostatistical Sciences, Winston Salem, NC, USA; 3 University of Otago, New Zealand; 4 School of Medical Sciences and Bioinformatics Institute, University of Auckland, New Zealand; 5 National University of Singapore, Yong Loo Lin School of Medicine, Department of Pathology, Singapore; 6 Wake Forest School of Medicine, Division of Radiologic Sciences, Center for Bioinformatics and Systems Biology, Winston Salem, NC, USA; 7 Sidra Medical and Research Center, Qatar; 8 Wake Forest School of Medicine, Department of Cancer Biology, Winston Salem, NC, USA; 9 Michigan State University, Department of Physiology, East Lansing, MI, USA; 10 Sidra Medical and Research Center, Division of Translational Medicine, Qatar

## 

**Figure 1:**
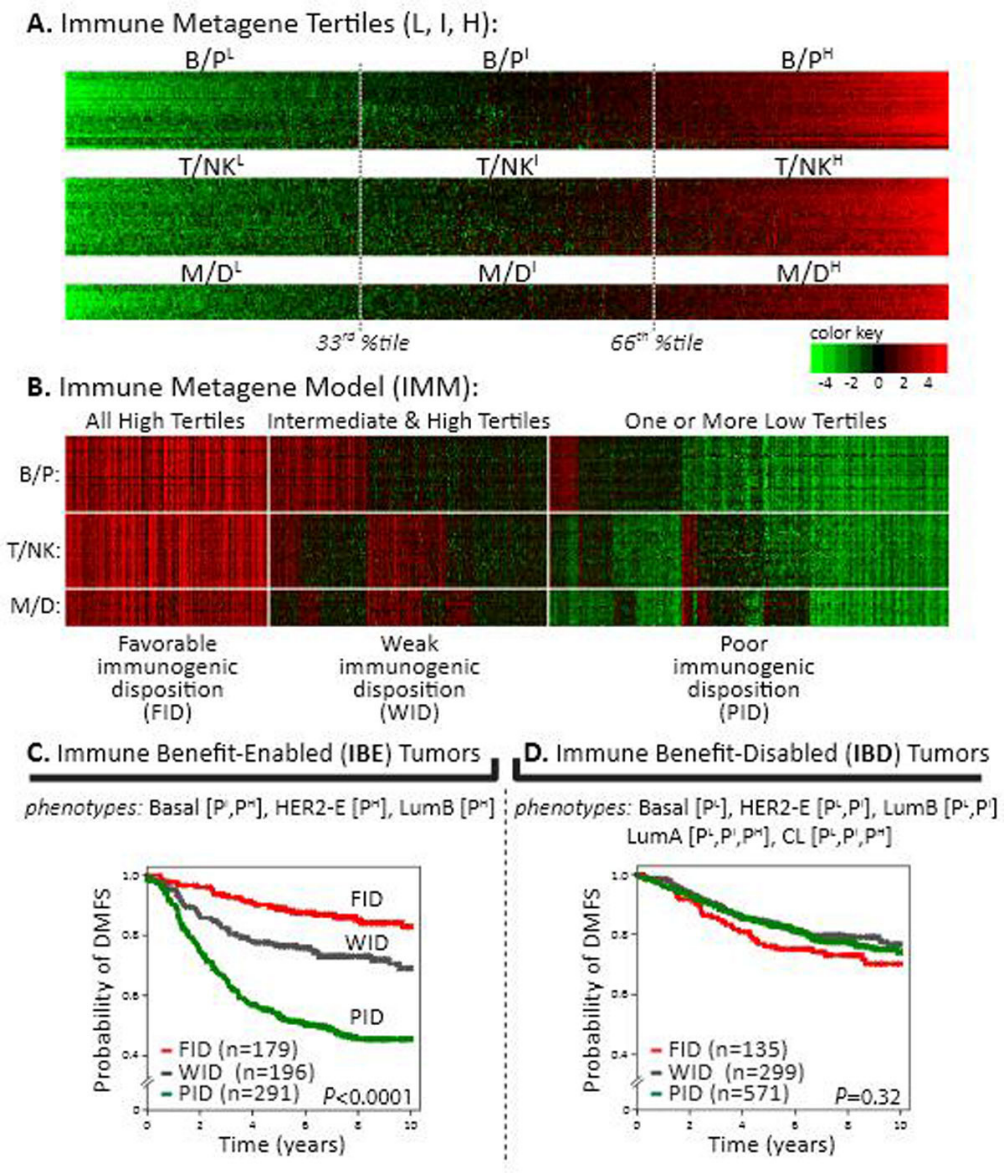
**(A-D) The immune metagenes are prognostic of IBE but no IBD breast cancer**. **(A) **Heatmaps of metagene expression levels (rows) across 1,954 tumors (columns). Key shows color scale of mean centered, log2-transformed gene signal intensities. For each metagene, tumors are aligned by ascending metagene scores; tertile thresholds are show (33^rd ^and 66^th ^percentiles) for defining low (L), intermediate (I) and high (H) metagene tertiles. **(B) **IMM prognostic risk groups are shown. **(C, D) **IBE and IBD type tumors are shown stratified by IMM subclasses (FID, WID, PID) in Kaplan-Meier plots of DMFS. The number of tumors (n) in each subclass is shown; the log-rank p-value is reported.

The abundance and functional orientation of tumor-infiltrating effector cells has long been observed to predict for reduced incidence of clinical metastasis and cancer-specific death. Using bioinformatics to mine large breast tumor microarray datasets, we and others have identified prognostic immune gene signatures, or *metagenes*. Robust evidence indicates that these metagenes are: 1) positively correlated with distant metastasis-free survival (DMFS) of patients, 2) comprised of genes that regulate immune cell-specific biology, and 3) reflective of the relative abundance of discernible populations of tumor infiltrating leukocytes. In recent work we have leveraged the statistical associations between the immune metagenes and the DMFS of breast cancer patients to explore the underlying phenotypes that differ in their ability to potentiate long-term, immune-mediated tumor rejection. Using a tumor classification model that combines the prognostic attributes of three distinct immune metagenes, termed the B/P, T/NK and M/D metagenes, we have identified molecular subtypes of breast cancer that either permit or prohibit prognostication by the immune metagenes. On this basis, we have delineated the phenotypic attributes of breast cancer that distinguish two novel immunogenic tumor subtypes, which we have defined as: *immune benefit-enabled *(IBE) and *immune benefit-disabled *(IBD). Phenotypically, IBE tumors comprise of Basal-like tumors and highly-proliferative HER2-Enriched and Luminal-B subtypes, while IBD tumors comprise of Claudin-Low, Luminal-A, and low-proliferative HER2-Enriched and Luminal-B tumors. Prognostically, IBE tumors (n = 666) can be stratified by the immune metagene model into prognostic subgroups with high statistical significance (*P*<0.0001,log-rank test), while IBD tumors cannot (n = 1005, *P *= 0.3) consistent with the capacity for an innate anti-tumor immunity against IBE tumors, but not IBD tumors, that guards against distant metastasis. Furthermore, these observations were independent of adjuvant treatment, and may owe to differential activation of immunomodulatory pathways. Network analysis revealed that IBE/IBD differentially-expressed genes (q<0.01) underlie highly-significant pathway activation scores for TGF-beta signaling in IBD (p < 0.0001), and Interferon-gamma signaling in IBE (p < 0.0001). Furthermore, 15 of 19 genes comprising the previously described *Immunologic Constant of Rejection *(Marincola and colleagues) were significantly overexpressed in IBE tumors (*P*-value range: 0.05-3.5E-14). Thus, we conclude that breast tumors can be dichotomized into two subtypes fundamentally distinct with respect to their potential for metastasis-protective immune responsiveness. These findings indicate new contexts for studying anti-tumor immunity and oncogenic mechanisms of immunosuppression in breast cancer. Whether IBE and IBD subtypes represent clinically-relevant contexts for assessing patient prognosis or evaluating the efficacy of immunotherapeutic treatments warrants further investigation (See Figure 1).

